# Effect of the rd1 mutation on motor performance in R6/2 and wild type mice

**DOI:** 10.1371/currents.RRN1303

**Published:** 2012-03-06

**Authors:** Liliana Menalled, Bassem F. El-Khodor, Monica Hornberger, Larry Park, David Howland, Dani Brunner

**Affiliations:** ^*^Senior Principal Scientist at PsychoGenics, Inc.; ^†^Senior Research Scientist, Pfizer Inc.; ^‡^Data Manager at Psychogenics, Inc; ^§^Director, PreClinical Research, CHDI Foundation, Inc; ^¶^Director of In Vivo Biology, CHDI Foundation Inc and ^#^Senior VP Behavioral R&D at PsychoGenics, Inc.

## Abstract

Homozygosis for the rd1 mutation in the Pbe6b gene results in the loss of the rod beta-subunit of the cyclic GMP phosphodiesterase and, eventually, of all rod and cone photoreceptors. The R6/2 mouse line is a widely used model of Huntington’s disease (HD). The original line was made available on a mixed background obtained by crossing, via ovarian transplant, female R6/2 (on a B6CBA mixed background) with male B6CBAF1/J mice. As the CBA/J strain used in the US is homozygous for the rd1 mutation and the breeding scheme does not ensure heterozygosis for the mutation, a significant percentage of the offspring on this mixed background is expected to be homozygous for the rd1 mutation. We investigate here the effect of rd1 homozygosis on motor function and examined the effects of the mutation on the R6/2 phenotype. Homozygosis for the rd1 mutation resulted in increased activity in the open field test and reduced rotarod test performance. In addition, rd1 mutation absence or heterozygosis reduced the differences between the R6/2 and the WT mice. Our recommendation for the neurodegeneration field, and for all mouse studies in general, is to carefully control homozygosis for retinal degeneration mutation, even when using tests of motor function.

## **Introduction**

The genomic revolution brought a myriad of new mouse models to the study of human processes in health and disease. In parallel to those models generated through targeted genetic manipulations, a large number of wild type (WT) mouse lines have been available thanks to devoted mouse breeders who selected and bred special qualities for representation in inbred lines. Some spontaneous mutations were bred to homozygosis intentionally whereas others were selected by accident. 

One common spontaneous mutation is the rd1 recessive mutation in the Pde6b gene that results in the disruption of the gene encoding the rod beta subunit of the cyclic GMP phosphodiesterase [1]. Mice homozygous for the rd1 mutation develop photoreceptors prenatally, lose all rod photoreceptors after the second postnatal week, and then suffer a slower loss of cone photoreceptors [2].

 For the study of the behavioral consequences of gene manipulation or other treatments, it has been an accepted wisdom to avoid strains homozygous for the rd1 mutation, especially in the area of cognition as many of the cognitive tests used with mice rely to some extent on visual information [3]. 

 We have established motor tests to study motor function in animal models of Huntington’s disease (HD) and have used for several years, as many other laboratories around the world have, the R6/2 mouse line on a mixed background obtained by crossing, via ovarian transplant, the female R6/2 (on a B6CBA mixed background) back to male B6CBAF1/J mice. As the CBA/J strain used (Jackson Laboratories Catalog No: 000656) is homozygous for the rd1 mutation and the breeding scheme does not ensure heterozygosis, a significant percentage of the offspring on this mixed background is expected to be homozygous for the rd1 mutation (and for any other parental recessive allele). 

 We investigate here the effect of rd1 homozygosis on performance in tests of motor function, using the R6/2 model as a prototypical model of motor deficits. We also considered the possibility that the pathology seen in the R6/2 model is affected by the rd1 mutation homozygosity and results in diminished or exaggerated motor deficits. 

##  **Materials and Methods**

## **Subjects**

R6/2 transgenic mice carrying the N-terminal region of a mutant human Huntington gene [4] were used in this study. Mice were bred in our colony by crossing ovarian transplanted WT females carrying ovaries from R6/2 females (Jackson Laboratories Catalog No: B6CBA-Tg(HDexon1)62Gpb/1J, Stock No. 002810) with B6CBA F1 wild-type males (Jackson Laboratories Catalog: B6CBAF1/J, Stock No. 100011). Mice were identified before weaning by real-time PCR of tail snips. In mutant mice, the CAG repeat length was analyzed by ABI 377 sequencer (Laragen Inc., CA) [4]. Mean CAG for the experimental subjects was ~106 and range was 14. Rd1 genotyping was done by Laragen Inc, CA using a protocol that detects the XMV-28 insertion. Around 30% of the offspring were homozygous for the mutation. 

Mice were weaned at approximately 3 weeks of age. Animals from multiple litters, with 3 or more pups per litter, were used for each treatment group (n=12; half of each gender). Mice were housed 4-5 mice per cage. In each cage, 2 wild-type mice of the same gender, but from different litters, were included in an attempt to provide normal social stimulation. Body weights of the experimental mice were recorded biweekly, and survival status monitored daily. All mice were housed in Optimice cages with wood shavings, food, and water; an enriched environment was created with the addition of play tunnels, shredded paper, and plastic bones. Breeder mice also received cotton nestlets and igloos instead of play tunnels. Mice had free access to regular food and water and, in addition, received wet powdered food placed inside a cup on the floor of the cage. This additional food was replaced fresh daily and started from weaning.  

Animal care was in accordance with the United States Public Health Service Policy on Humane Care and Use of Laboratory Animals, and procedures were approved by the Institutional Animal and Use Committee of Psychogenics, Inc. (PHS OLAW animal welfare assurance number A4471-01), an AAALAC International accredited institution (Unit #001213).

**Experimental procedures**

Mice were allowed to acclimate to the experimental room for at least one hour prior to the beginning of any experiment. Mice were transported from the colony room to the experimental rooms in their home cages and returned to the colony as soon as the behavioral testing was completed. Animals were always tested during the light phase of the diurnal cycle.

### **Open field**

At 4, 6, 8 and 12 weeks of age mice were placed in the center of the activity chambers (Med Associates Inc, St Albans, VT; 27 x 27 x 20.3 cm) equipped with IR beams, and their behavior was recorded for 30 min. Quantitative analysis was performed on the total locomotion and total rearing frequency. 

 **Rotarod**

Mice were tested over two consecutive days at 4 weeks of age and retested over one day at 6, 8 and 10 weeks of age. Each daily session included a training trial of 5 min at 4 RPM on the rotarod apparatus (AccuScan, OH). One hour later, the animals were tested for 3 consecutive accelerating trials of 5 min with the speed changing from 0 to 40 RPM over 360 s and an inter-trial interval at least 30 min. The latency to fall from the rod was recorded. Mice remaining on the rod for more than 360 s were removed and their time scored as 360 sec. In less than 1% if trials, a fecal bolus dropping broke the infrared beam and terminated the trial prematurely, in which case data was not used for analysis.

 **Grip strength**

Grip strength was assessed at 12-13 weeks of age. Mice were held by the tail and lowered towards the mesh grip piece on the push-pull gauge (Ugo Basile, Italy) until the animal grabbed it with both front paws. The animal was then lowered toward the platform and gently pulled backwards with consistent force until it released its grip. The forelimb grip force was recorded on the strain gauge. After testing animals were placed back into home cage.

### **Survival**

Mice were examined daily in order to determine lifespan and were considered to have died when they no longer had a heartbeat.

 **Other experimental details**

Mice in this study were also planned as controls for a drug study and thus received daily oral gavage (p.o., volume injection of 5 ml/kg) of water. Half of the mice underwent cognitive testing (fear conditioning and swim tank); other tests (pole testing and rearing climbing) were also performed but the results are not included here. Cognitive testing history had no effects on the results shown here.  **Data analysis**

Repeated measures analysis of variance (ANOVA) was carried out with SAS (SAS Institute Inc.) using Mixed Effect Models. This approach is based on likelihood estimation which is more robust to missing values than moment estimation. The models were fitted using the procedure PROC MIXED (Singer, 1998). HD genotype, rd1 genotype, age and their interactions were considered in the model. We pooled the results of WT and heterozygous mice for the rd1 mutation. Significant interactions were followed up with simple main effects to find at which age the genotype differences reached significance.  Survival was assessed using Kaplan-Meier analysis. An alpha level of .05 was selected for all inferential statistics. 

##  **Results**

## **Open field**

Homozygosis for the rd1 mutation was associated with increased distance covered at all ages in both R6/2 and WT groups, although the effect was more pronounced in the latter (rd1 Genotype and rd1 Genotype x HD Genotype interaction: Fs(1,62)> 17.9; ps < .0001; Fig. 1). Rd1 homozygosis also resulted in increased rearing in the WT mice, but not in the R6/2 mice, starting at 6 weeks of age (HD Genotype x rd1 Genotype x Age interaction: F(3,183)=4.56, p<.002). 

**Figure:**
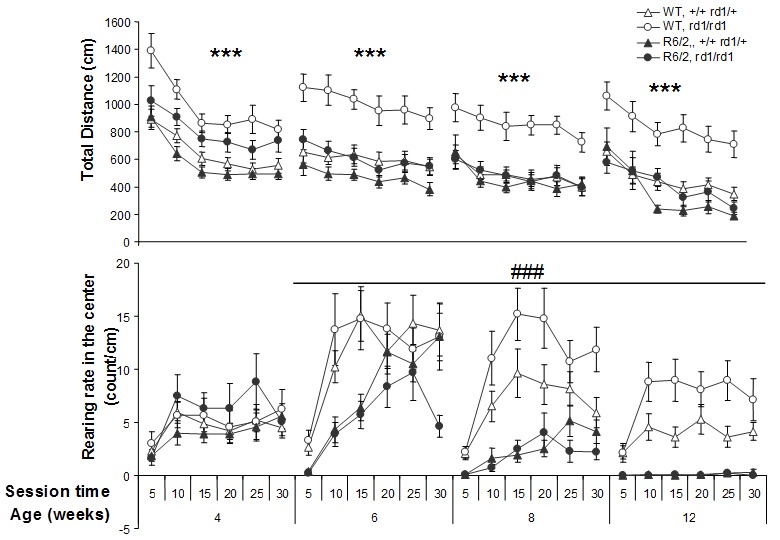
 **Figure 1.** Performance in the Open Field of R6/2 mice with and without homozygosis for the rd1 mutation. Top: total distance; Bottom: Rearing rate in the center. *** Significant hyperactivity observed in rd1 homozygous mice (p < 0.0005). ### Significant increase in rearing in the center observed in rd1 homozygous mice from the WT group (p < 0.0005).

### **Open field**

Homozygosis for the rd1 mutation was associated with increased distance covered at all ages in both R6/2 and WT groups, although the effect was more pronounced in the latter (rd1 Genotype and rd1 Genotype x HD Genotype interaction: Fs(1,62)> 17.9; ps < .0001; Fig. 1). Rd1 homozygosis also resulted in increased rearing in the WT mice, but not in the R6/2 mice, starting at 6 weeks of age (HD Genotype x rd1 Genotype x Age interaction: F(3,183)=4.56, p<.002).       

###     **Rotarod**

Homozygosis for the rd1 mutation was associated with a decreased latency to fall in the rotarod test, reaching significance only at 6 weeks of age in the WT mice (HD Genotype x rd1 Genotype x Age interaction and post hocs: Fs>5.3, p<.01; Fig. 2).

**Figure:**
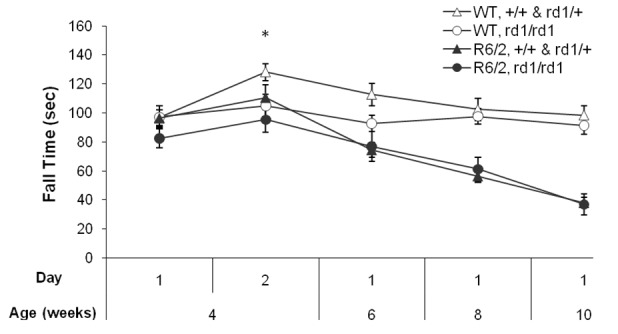
***Figure 2*** . Performance in the rotarod test of R6/2 mice with and without homozygosis for the rd1 mutation. * Significantly decreased rotarod performance of homozygous rd1 mice from the WT group (p < 0.05).

###      **Grip strength**

The rd1 mutation had no effect on grip strength at 12-13 weeks of age. Male and female R6/2 showed reduced grip strength (HD Genotype effect: p < 0.05; not shown).  

     **Survival**

The rd1 homozygosis had no effect on survival. R6/2 mice showed the typical reduced survival with median about 130 days (not shown). 

     **Body weight**

Rd1 homozygosis had no effect on body weight in either WT or R6/2 mice. Both male and female R6/2 mice showed the typical body weight loss starting at around 7 weeks of age (HD Genotype x Age interaction and post-hocs: Fs > 6.0, ps < 0.02; not shown). 

##  **Discussion** 

     We have demonstrated that homozygosis for the rd1 mutation results in large increases in activity in the open field test and decreased performance in the rotarod test. This was a surprising result, given that these are tests used for assessment of activity (open field) and motor function (rotarod) for which visual acuity is not assumed to be of great importance.  In the specific case under study, removing the rd1 effects reduced the HD genotyping differences, as the effects of rd1 homozygosis were more pronounced in the WT mice (increasing their behavior) than in the R6/2 mice.   

     The R6/2 mouse line, which is used by many labs in the US, is also commercially available at Jackson although with a slightly larger CAG repeat length (~120) than the one used in the present study (~106). As we noted that the CAG repeat length in our experimental subjects was becoming progressively shorter, Jackson Laboratories recreated the original line from cryopreserved embryos and renamed it (Catalog No: B6CBA-Tg(HDexon1)62Gpb/3J, Stock No. 006494. This strain is also referred to as CHDI-80000001-4 and R62(CHDI-001-4)). Fertilizing the R6/2 transgenic eggs with B6CBAF1/J sperm results in a random mix of genetic contributions from both parental strains which results in neither an F1 (perfect balance of parental alleles) nor an F2 (balanced randomization of parental alleles). Instead, the resulting mix can fluctuate greatly from generation to generation depending on the particular genetic composition of the breeders. Thus, if the eggs happen to be homozygous for the rd1 mutation, then up to 50% of the offspring will be homozygous for rd1, after crossing with B6CBAF1/J males. Cohorts of R6/2 mice bred this way will have an unpredictable number of homozygous rd1 individuals, and whole colonies could drift towards or against homozygosis. (This applies, of course, to all recessive genes, not just the rd1 mutation.) We have, in all studies conducted after this assessment of rd1 effects, removed the rd1 homozygosis using different techniques: a) genotyping and excluding rd1 homozygous mice, b) used the B6CBAF1/J males created with the CBA/CaJ line (Catalog No: 000654) which does not carry the rd1 mutation, or c) used C57BL/6J males (Stock No: 000664) for fertilization of R6/2 eggs.   

     Retinal degeneration has effects on regions of the brain associated with light entrainment [5] and can potentially affect circadian and other functions. Therefore, our findings regarding activity and motor function maybe directly due to vision deficits or due to changes in other systems.   

     The reasons for the differential effects of rd1 on WT and R6/2 mice are not clear, but could relate to the beneficial effects of BDNF on rd1-related retinal pathology [6]. As the R6/2 mice have deficits in BDNF [7], their own HD-related pathology may render them insensitive to the additive deleterious effects of rd1 homozygosis. Consistently, R6/2 mice retinal pathology [8] and circadian deficits [9] maybe related to reduced light entrainment.   

     Our recommendation for the neurodegeneration field, and for all mouse studies in general, is to carefully control homozygosis for retinal degeneration genes. The suggestion to avoid homozygosis for recessive genes in general, is not new for the HD field (see the Hereditary Disease Foundation workshop in Cardiff, Wales July 2002: "Behavioral Assessment of Mouse Models of Huntington’s Disease"; http://www.hdfoundation.org/). In turn, the suggestion to avoid the rd1 mutation in particular, is not new for cognitive experimental psychologists. Our results extend the recommendation to exclude homozygosis of genes causing visual deficits even when studying motor function in HD and other indications. In addition, our results highlight the perilous and unpredictable nature of gene-gene interactions and their effects of pathological phenotypes.  

## **Competing Interests**

CHDI Foundation is a not-for-profit biomedical research organization exclusively dedicated to discovering and developing therapeutics that slow the progression of Huntington’s disease. CHDI Foundation conducts research in a number of different ways; for the purposes of this manuscript, all research was conceptualized, planned, and directed by all authors listed and conducted at PsychoGenics, Inc., a contract research organization.  

CHDI Foundation provides financial support for PLoS Currents: Huntington Disease. Editorial responsibility for all content remains entirely within the remit of the Public Library of Science, the Editors, and Board of Reviewers. 

At the time the study was conducted, Menalled L, El Kodhor B, Patry M, and Brunner D were all employed by PsychoGenics, Inc., a for-profit institution. The authors have declared that no further competing interests exist.  

## **A** **cknowledgments**

We are grateful for technical assistance provided by Tierra Anderson and statistical support by Rich Mushlin.

## **Funding information**

This work was supported by the CHDI Foundation. 
